# Development of a plant conveyance system using an AGV and a self-designed plant-handling device: A case study of DIY plant phenotyping

**DOI:** 10.1270/jsbbs.21070

**Published:** 2022-02-17

**Authors:** Takanari Tanabata, Kunihiro Kodama, Takuyu Hashiguchi, Daisuke Inomata, Hidenori Tanaka, Sachiko Isobe

**Affiliations:** 1 Department of Frontier Research and Development, Kazusa DNA Research Institute, 2-6-7 Kazusa-kamatari, Kisarazu, Chiba 292-0818, Japan; 2 Faculty of Agriculture, University of Miyazaki, 1-1 Gakuenkibanadai-Nishi, Miyazaki 889-2192, Japan; 3 SuanaScience, 4-7-5 Sakurada, Kuki, Saitama 340-0203, Japan; 4 Interdisciplinary Graduate School of Agriculture and Engineering, University of Miyazaki, 1-1 Gakuenkibanadai-Nishi, Miyazaki 889-2192, Japan

**Keywords:** plant phenotyping, convey system, automation, AGV, DIY

## Abstract

Plant phenotyping technology has been actively developed in recent years, but the introduction of these technologies into the field of agronomic research has not progressed as expected, in part due to the need for flexibility and low cost. “DIY” (Do It Yourself) methodologies are an efficient way to overcome such obstacles. Devices with modular functionality are critical to DIY experimentation, allowing researchers flexibility of design. In this study, we developed a plant conveyance system using a commercial AGV (Automated Guided Vehicle) as a case study of DIY plant phenotyping. The convey module consists of two devices, a running device and a plant-handling device. The running device was developed based on a commercial AGV Kit. The plant-handling device, plant stands, and pot attachments were originally designed and fabricated by us and our associates. Software was also developed for connecting the devices and operating the system. The run route was set with magnetic tape, which can be easily changed or rerouted. Our plant delivery system was developed with low cost and having high flexibility, as a unit that can contribute to others’ DIY’ plant research efforts as well as our own. It is expected that the developed devices will contribute to diverse phenotype observations of plants in the greenhouse as well as to other important functions in plant breeding and agricultural production.

## Introduction

Plant phenotyping technologies are used to evaluate the quantitative and qualitative traits of plants and their sequential changes during growth. Diverse plant species are targeted in plant science, breeding, and agriculture, and the required phenotyping technologies are as diverse as the targeted plants. A significant number of reports on new digital phenotyping technology have been published in recent years, suggesting that this research field is recognized as important (Costa *et al.* 2019, Fahlgren *et al.* 2015, Mishra *et al.* 2016, Tardieu *et al.* 2017). In crop breeding, large numbers of plants are compared so that the best individuals can be selected. Therefore, it is necessary to build a high-throughput system that can acquire a large amount of data by combining digital phenotyping technologies with automation technologies.

In the case of digital plant measurement technologies using relatively large measurement devices, it is necessary to either deliver the target plants to the measurement device or move the measurement device itself. Various devices for automatic measurement have been developed to meet these demands (Humplík *et al.* 2015, Li *et al.* 2021). Automated imaging systems that combine digital imaging technology with transport devices have been developed for indoor settings (Clauw *et al.* 2015, Fujita *et al.* 2018, Granier *et al.* 2006, Ishizuka *et al.* 2005, Tisné *et al.* 2013), for greenhouses (LemnaTech 2021, Reuzeau *et al.* 2006), and for the field (Bai *et al.* 2016, Friedli *et al.* 2016, Vadez *et al.* 2015, Virlet *et al.* 2016); in addition, specialized imaging devices for root measurement systems have been developed to be adaptable to the measurement purpose and cultivation location (Yazdanbakhsh and Fisahn 2009).

Two tasks are frequently required in plant phenotyping: conveyance and measurement. In a high-throughput phenotyping system, these tasks should be automated. For conveyance, most high-throughput phenotyping systems use conveyor belts to transport plants from the cultivation area to the measurement area, e.g., the image acquisition site (LemnaTech 2021, Reuzeau *et al.* 2006). Until recently, measurement systems involving the conveyance of plants or plant measurement devices have most often been designed to fit specific conditions, such as a particular installation site (greenhouse or field of a particular size/layout), a particular size and/or shape of plant, a particular number of plants, and/or particular cultivation conditions. In plant conveyance systems using belt conveyors, multiple belt conveyor units are connected and laid out in a greenhouse. The layout of the cultivation and the measurement area is fixed, and belt conveyor units are set and controlled with consideration of the size of the delivered objects. In addition to the physical parts, the control program must be optimized to fit the conditions of the system. The optimal layout of plant pots in the greenhouse depends on the purpose of the trial and the size of the target plants. Frequent change of layout is not realistic as it would require a high level of expertise and cost (Table 1). Because of the large installations, operational and maintenance costs of several million dollars, the use of conveyor belt systems has been limited to only a few research institutions.

Meanwhile, different conditions and settings are required for each plant phenotyping research. For example, Nahar and Ikeda (2002) investigated effects of figaron and water deficit on soybean seed yield. Tow soybean cultivars were grown in the greenhouse with four different water conditions × absence or presence of figaron treatment. In this case, a total of 64 matured soybean plants in five-liter plastic pots were arranged in the four replications. Meanwhile, Fatichin *et al.* (2013) investigated early vegetative growth of 27 soybean varieties. In this trial, a total of 1,350 (27 varieties × 10 pots × 5 replications) of young soybean seedlings were grown in the plastic pots containing 500 g soil in the greenhouse until 28 days after sowing. Both studies observed soybean growth in the greenhouse, however, the number of pots, pot sizes and layout of the trials were different. To respond effectively to such differences, flexibility in the transport system is arguably the most desirable design feature. Use of autonomous mobile robots is an effective approach, and systems that can be customized without changing the hardware have been proposed (Xiang *et al.* 2020). Drones are the most popular type of autonomous mobile robot, and they have been frequently used in plant phenotyping (Guo *et al.* 2021). However, the use of drones is still difficult in situations where GPS signals cannot be received, as in many greenhouses. In addition, the device weight and flight time of the measurement systems are limited due to power supply issues.

The introduction of digital phenotyping technologies into horticultural/agronomic research and practice has not progressed as expected, conventional phenotyping methods have still been used in many plant breeding programs and research fields. Multiple factors might be causing the delay in technology adoption. Of these, the two most critical factors are likely flexibility and cost. The former arises from the fact that the required plant measurement techniques vary depending on the plant species, the target to be measured, and the intended use of the measured data; however, many newly developed phenotyping technologies are only applicable under a narrow range of conditions. The latter arises from the fact that technologies specialized for specific purposes are not suitable for mass production, and given the small number of users, they are inevitably expensive.

In recent years, there has been a movement in the field of scientific research to develop experimental infrastructures based on the “DIY” (Do It Yourself) concept. This is thought to be the antithesis of another recent trend whereby scientific research is performed in large-scale projects under the assumption that high-impact research can only be conducted by amply funded research groups (Ravindran 2020). Along with making science more democratic generally, introducing DIY methods and technologies into research helps improve flexibility and cost, the two main factors hindering the adoption of digital plant trait assessment (May 2019). In fact, the merits of the DIY concept have been mentioned in several recent studies of plant digital phenotyping technologies (Dobrescu *et al.* 2017, Panjvani *et al.* 2019, Wang *et al.* 2020).

Hence, we developed an automated system to deliver plant pots back and forth from their cultivation areas into a specified phenotype measurement area that is equipped with cameras and computers. The system for DIY plant phenotyping was developed to be modular and is intended to be used in a greenhouse or a room with a relatively large area. A commercial AGV (Automated Guided Vehicle) kit was used as an autonomous robot; as this feature was generic, its use allowed us to reduce the overall cost of development. In this study, we report the specification of the developed plant conveyance system and the concept of DIY plant phenotyping. We further describe our philosophy for the development of DIY plant phenotyping technologies in the Discussion section, based on the principle that open discussion of a philosophy or concept is sometimes critical to advancing science into new areas.

## Materials and Methods

**Fig. 1.**
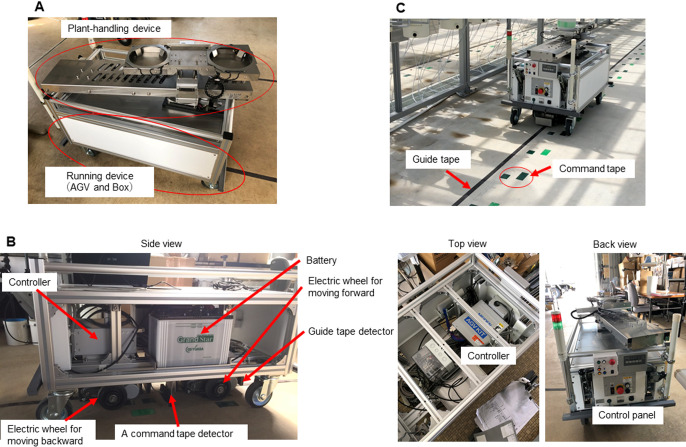
The automatic plant conveyance vehicle. A) Running and plant-handling devices. B) Hardware provided by the AGV kit. C) Guide and command tapes.

The plant conveyance module consists of two devices, a running device and a plant-handling device (Fig. 1A). A commercial AGV Kit, Meiden AGV Kit (MK2/5 Series), was used for the running device (MEIDENSHA Corp., https://www.meidensha.com). The AGV automatically moves along a path of magnetic tape installed on the floor. The reasons for choosing AGV technology was the wide availability of commercial kits, the capacity of commercial AGVs to carry heavy loads, and their flexibility in running routes. The AGV kit used in this study can carry a load as heavy as 700 kg. Rerouting is easily accomplished by resetting the magnetic tape on the floor, depending on the trial design. The AGV kit includes hardware and software packages for controlling the vehicle: i.e., electric wheels, a controller, guide tapes, a command tape detector, guide tape detectors, a control panel, and a battery. A box-like body with aluminum frame to cover the AGV was created for mounting the hardware parts to control the AGV. A microcomputer, Raspberry PI 2, was also included in the box as a relay station between the AGV controller and the operation PC via Wi-Fi. Another role of the box is to protect the AGV from the dust and water dispersed from the plant pots.

The plant-handling device was created from scratch as there were no commercial products available to adapt to this purpose. The device consists of a base to attach to the AGV, a stage on which the pots are placed, four motors for moving the tables in different directions (rotation, up and down, left and light, forward and back), a sensor to detect the correct pot position, and motor controllers. The program controlling the movements of the device was created with C (GCC on Linux Raspbian stretch-lite 4.14.62-v7) and installed onto a microcomputer (Raspberry PI2).

Separate programs for controlling the running route and for handling the specified plants were created with Visual C++ (Microsoft Corp., https://www.microsoft.com) and installed on the operation PC Endeavor NA512E (EPSON Corp., https://www.epson.jp). The programs are operated using a graphic user interface (GUI) so that the user can handle the system control remotely via Wi-Fi.

Dedicated metal pot stands were made for accommodating the plant-handling function of the developed system, set in the greenhouse at University of Miyazaki (width 16 m, depth 28 m, height 4 m). Metal pot adaptors were also created for hanging the pots on the pot stands. Soybean seeds of four varieties, Fukuyutaka, Misuzudaizu, Williams82 and Moshidou Gong 503, were sown in 1/5000a Wagner pots with burnt soil for testing the plant pot conveyance system. Irrigation was performed using a water tube placed on the pot stands.

## Results

### The running device

The aluminum frame of the running device was mounted to parts of the AGV kit, i.e., electric wheels, a command tape detector, magnetic guide tape detectors, a battery, an AGV controller, an AGV control panel, and a microcomputer (Fig. 1B). The AGV senses magnetism from the magnetic tape placed on the floor, receives commands, and performs a programmed action. Two types of magnetic tape, guide and command, were used (Fig. 1C). The two tapes have two separate roles: the guide tape delineates the path over which the AGV moves while the command tapes issue stop and turn commands and are placed on start/stop or junction points for branching routes. The command tapes can be set in multiple patterns and were assigned specific IDs using the program provided by the MEIDENSYA kit. Assignment of movements (i.e., stop or turn) for each ID was set by the program in the operation PC. Details for the setting assignments are described in the system control section.

### Hanging pot stands and plant-handling device

**Fig. 2.**
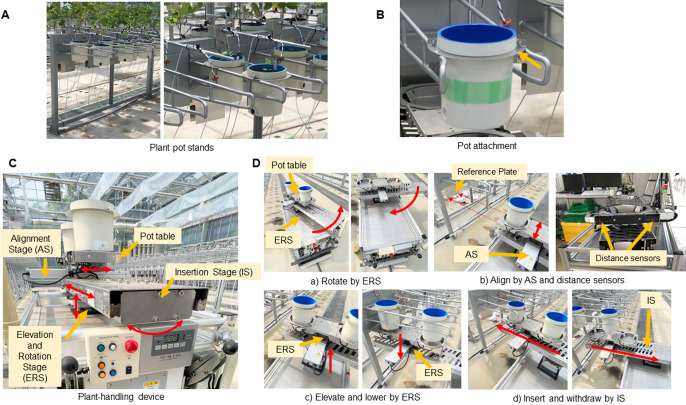
Photos of plant pot stands and the plant-handling device. A) Plant pot stands, B) A pot attachment, C) Four main components of the plant-handling device, D) The actions required to return the pots. a) Rotate the pot table by ERS, b) Align the pot table by AS and distance sensors, c) Elevate and lower the pot table by ERS, d) Insert and withdraw the pot table by IS.

In order to transfer a plant pot from the cultivating place to the running device automatically, we designed and developed a plant-handling device and pot hanging stands. Metal stands for the greenhouse were built to hold plant pots during cultivation, to accommodate the mechanisms of the plant-handling device (Fig. 2A). A pair of metal arms supports the two 1/5000 Wagner pots. The ends of the arms are tapered outward to guide the insertion and removal of the pots. An attachment was mounted on each pot to hold it on the arms of the stand (Fig. 2B).

The plant-handling device was developed for automatically picking up and returning the plant pots to the stand arms. The device consisted of four main components, i.e., the pot table, the insertion stage (IS), the alignment stage (AS) and the elevation and rotation stage (ERS), and it can hold two 1/5000 Wagner pots at once on the motorized stage (Fig. 2C). Two pots are put on the pot table, and the pot table is moved by the three stages with four actions, insert/withdraw by IS, align position by AS and rotate and elevate/lower by ERS.

When the device is picking up the pots or returning them to the pot stand, the AGV runs parallel along the pot stand to the picking-up position. The AGV turns at the stop position so that the body is facing the pot stand, and stops at specified position an error range of plus or minus 15 mm. Then, the ERS rotates the pot table 90 degrees to the direction of the AGV body (Fig. 2Da). The pot table position is subsequently aligned by AS to the pot position so that the stage can be inserted at an exact position under the stand arms (Fig. 2Db). When the AGV returns the pots to the stand arms, the pot table is raised by ERS to the height where the pots can simply “float” onto the plant-stand arms (Fig. 2Dc). Then, the pot table is inserted under the stand arm by IS and lowered by ERS. (Fig. 2Dd). The reverse movements are performed for picking up the pots.

**Fig. 3.**
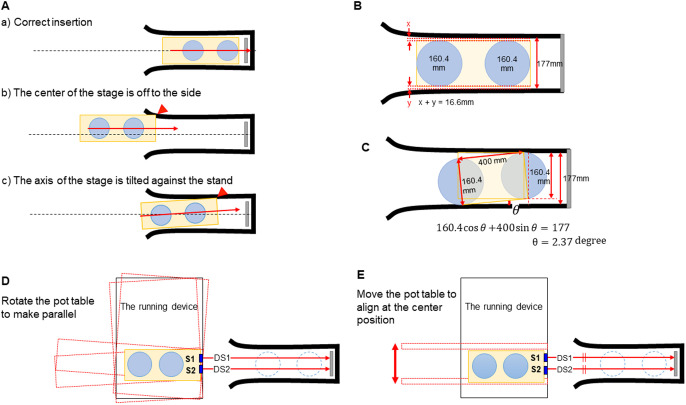
Alignment of the pot table position to insert the pot table at the right position. A) Three cases of pot table insertion. a) Correct insertion, b) The center of the stage is off to the side, c) The axis of the stage is titled against the stand. B) Gap distances between the pot table and the hanging arm. C) Error range of the tilt angle of the pot table against hanging arm. D) Align angle of the pot table. E) Align center position of the pot table. Yellow squares, Pot table; blue circles, plant pots; thick black lines, hanging arm; gray bars, reference plate; blue squares, distance sensors; dotted lines, center line of hanging arm; red arrows, moving direction of the pot table.

In order to insert the pot table under the stand in the normal position, the center lines of the pot hanging arm and the pot table must be matched (Fig. 3Aa). If the center line of the pot table is shifted sideways (Fig. 3Ab) or tilted (Fig. 3Ac), it cannot be inserted in the normal position. The total length of the gap between the hanging arm and the pot table is 16.6 mm, and the pot table must be inserted under the hanging arm within the width error range of less than 16.6 mm (Fig. 3B), and tilt angle error range of less than 2.37 degrees (Fig. 3C). The error range of the stopping position of the AGV is plus or minus 15 mm. In addition, it is difficult to put the magnetic tape that specifies the stop position of the AGV at the absolute position. That is, the accuracy of the stopping position of the AGV is not sufficient for the requirement of the stage insertion position. Therefore, we developed a function to adjust the pot table position for the case that the stopping position of the AGV is slightly off.

Two distance sensors were mounted in front of the pot table to sense the position of the pot table relative to the hanging arm, and a stainless plate was mounted at the back of the hanging arm to serve as a reference position (Fig. 3D). After the AGV stops, two distance sensors (S1, S2) measure the distances (DS1, DS2) between the pot table and the reference plate. The angle *θ* is then obtained from the distance between DS1, DS2 and the distance (D) between the positions of S1 and S2.

$$\theta =\tan^{-1}\left(\frac{DS1-DS2}{D}\right)$$

The pot table is rotated by ERS to the angle *θ* so that the insertion direction is parallel to the hanging arm (Fig. 3D). Then, AS moves the pot table so that DS1 and DS2 are the same length to align the center of pot table to that of the hanging arm (Fig. 3E).

### System control

**Fig. 4.**
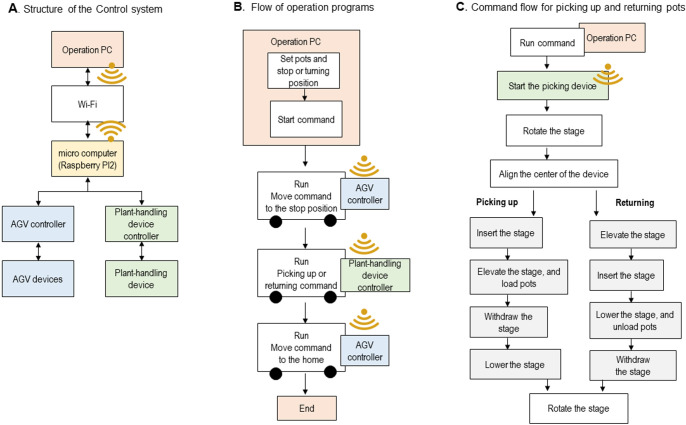
An overview of the structure of the control system and the commands it follows. A) Structure of the control system, B) A flow-of-operation program, C) The command flow for picking up and returning pots.

The running route and selection of plants were controlled from the operation PC (Fig. 4A) via Wi-Fi. In this study, we used a PC (EPSON Endeavor NA512E) with the following specs: CPU Intel i7-6500U, 8 GB memory, equipped with a Wi-Fi device. The minimum spec PC required for running the operation program is sufficient to run Windows 10 OS. A microcomputer, Raspberry PI2, was used as the relay station between the AGV controller and the operation PC. The Raspberry PI2 receives commands from the operation PC and forwards them to the microcomputers in the AGV and handling device controllers. The program in the AGV microcomputers, i.e., provided as a software package in the controller, was originally provided by MEIDENSHA Corp. We added to it the program sending the commands to the AGV controller. Other software programs in the operation PC, i.e., the Raspberry PI2, were created originally for the system.

The flow of operating programs is shown in Fig. 4B. Unique IDs were given to each plant pot and position where the AGV was to stop or turn. The running route of the AGV was set by specifying the pots and position IDs, and assigning the stop or turning commands for each position ID. This setting was performed by the program in the operations PC. Then, the run command was sent from the operations PC to the AGV controller via the Raspberry PI2 and Wi-Fi. After the AGV arrived and stopped at the given position, the AGV controller sent a signal that the mission was completed. The next command was automatically sent from the operation PC to the plant-handling device controller for the sequential movements required for picking up or returning a plant (Fig. 4C). After the mission of the plant-handling device was finished, a report signal was sent to the control PC. Then the control PC sent a command to the AGV controller to back up the vehicle to the home position.

### Establishing a path of movement through the greenhouse

The developed system was installed in a greenhouse (width 16 m, depth 28 m, height 4 m) at the University of Miyazaki. Hanging pot stands were set up in the greenhouse for 400 plant pots. The layout of magnetic tape was changed several times in verifying the optimization of the driving route. The AGV moves at a maximum linear speed of 30 m/min, and the average distance between the home position and pots was 15 m. Therefore, it took an average of 1–2 minutes to move from the home position to the destination and back. In addition, approximately 80 seconds were required for the pot picking-up or returning operation. Therefore, it required a total of 3 minutes to convey a set of two pots to the measurement area. When the conveyance system is operated for six hours per a day, one module was able to deliver about 240 pots per day from the pot stands to the home position, and back to their pot stands. The developed operation system allows multiple AGVs to be controlled at the same time, so we installed two modules at Miyazaki University to increase the number of pots delivered per day to a maximum of about 480.

**Fig. 5.**
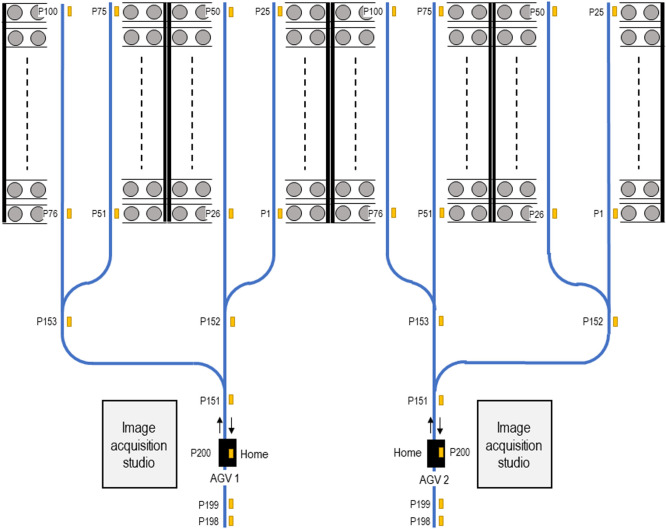
Layout of pot stands, pots, magnetic tapes and AGVs. Blue lines, guide tape layout; Yellow boxes, command tapes; gray circles, plant pots; solid black bars, pot stands; black boxes, AGVs. Numbers with P: Position IDs.

The greenhouse was divided into two areas for each module, and 100 plant pots were set in each area in four rows (Fig. 5). Guide tapes were placed on the floor along each row, shown as blue lines in Fig. 5. As shown by the orange tags in the Fig. 5, command tapes were placed on positions that were given a position ID and were assigned an instruction for which way the AGV should move. A total of 100 positions were set as pot positions and were given IDs from P1 to P100. P200 was assigned to the home position. Three positions, P151, P152 and P153, were placed on the transport path branches and were assigned turn commands. P198 and P199 were assigned as backyard positions for resetting the AGVs to the home position. The assignment of position ID for each pattern of command tape was performed using an application provided from the MEIDENSHA AGV kit.

In the operation tests, we set a moving speed of 10 m/min for this test. We succeeded in delivering the pots containing soybean plants from the pot stands to the home position (photo acquisition area) without any falls or drops. The maximum weight of the delivered soybean pot was approximately 10 kg. For pots placed at P50, it took approximately 5.5 minutes to complete the picking-up-pots mission: i.e., starting from home (P200), to pick up two plant pots, and return to the home position for measurement. The operation time included about 4 minutes for travelling, and 75 seconds for pot handling. When the target pot was placed at a closer position, for example P51, it took approximately 3 minutes to complete the mission. In this case, the operation time included about 100 seconds for traveling and 75 seconds for pot handling. The combinations of delivered pots was changed easily by editing the setting of the operation program.

The development of various devices has also led to increased extensibility of plant phenotyping technology. For example, at University of Miyazaki, image-acquisition studios that we developed were set up next to the home position of the AGV to take digital images for 3D modeling (Fig. 5). Currently, the plant pots are moved between the AGV and the studio by hand. However, if a fully automated system is needed, we could create a separate module to move the pots. The development of devices with various functions would give us more freedom to design a comprehensive custom-made system of plant phenotyping.

## Discussion

### Development of a plant conveyance system using an AGV

In this study, we report an automatic plant pot conveyance system for a greenhouse using AGV kits. Belt conveyor systems are currently the most frequently used type of plant conveyance system in the greenhouse (Table 1, Arend *et al.* 2016, Hairmansis *et al.* 2014, Klukas *et al.* 2014, Mazis *et al.* 2020, Pratap *et al.* 2015). The fee for development of a running and a plant-handling devices, including the cost of parts, fabrication work, and system design, were approximately 40,000 USD and 6,000 USD, respectively. It was not cheap, but it was less than 1/10^th^ of the cost of a plant-delivery system using belt conveyor units for delivering one hundred plant pots. Our system also has the flexibility to change the plant conveyance route simply by changing the layout of the magnetic tape, while significant cost is required to change the layout of a belt conveyor system. In addition, the number of carts used can be increased or decreased according to the number of cultivated pots to be conveyed. Having fewer mechanical parts especially in the passage through which the plants pass also contributes to lower cost and less chance of machine failure; in short, to increased flexibility and lower operation cost.

The plant-handling device and pot stands were newly designed and manufactured to suit our purpose. In order to keep the development cost low and to make the system work robustly, we invented a simple method to load and unload the pots on the pot handling-device by floating the pots above the pot hanging arm. In addition, the plant-handling device is equipped with the pot table alignment function using three motorized stages (AS, ERS and IS), distance sensors and a reference plate position, which allows the AGV to stop within a certain margin of error. With the function, plant pots could be automatically picked up or returned with robust behavior.

Xiang *et al.* (2020) reported a robotic assay for drought (RoAD) by using a mobile cart. In this system, measurement equipment was carried by a robot, and plants were not delivered. The approach of moving the measurement device in plant phenotyping has also frequently performed by using drones (Frangulea *et al.* 2021, Guo *et al.* 2021). Moving devices has benefit to require less cost of setting equipment, however, it often decrease measurement accuracy due to the difficulty in controlling the measurement environment. Our plant transport system is in the middle of blet conveyor systems and drones in terms of ease of installation and stability of transport, and is useful for measuring a large number of objects with high accuracy in a controlled environment such as in an image acquisition studio (Table 1).

### Definition of DIY plant phenotyping

The system in this study was developed not only to create a specific plant delivery system for a specific crop and greenhouse, but also with the objective of creating a device and software that enabled the design of other DIY phenotyping systems for other crops, greenhouses, or even fields. In this manuscript, we defined DIY phenotyping as “phenotyping with a system that is built or re-built by users with shared or available software and hardware”. The aims of DIY phenotyping are reducing the building cost and increasing the flexibility. The building cost includes costs of the parts, fabrication work, and system design. DIY phenotyping decreases the outside costs for fabrication and system design because users perform those tasks by themselves. Furthermore, the modular elements of the system can be reconfigured and reused when research groups’ needs change, reducing future costs. DIY phenotyping encompasses all the technologies and information involved in phenotyping, including the automation of cultivation management and sampling. For example, the purchase and use of IoT devices (Bagley *et al.* 2020) and the creation of systems using microcomputers and sensor components such as the Raspberry PI (Tovar *et al.* 2018) are considered DIY phenotyping activities.

### A plant conveyance system, as an item for DIY plant phenotyping

**Fig. 6.**
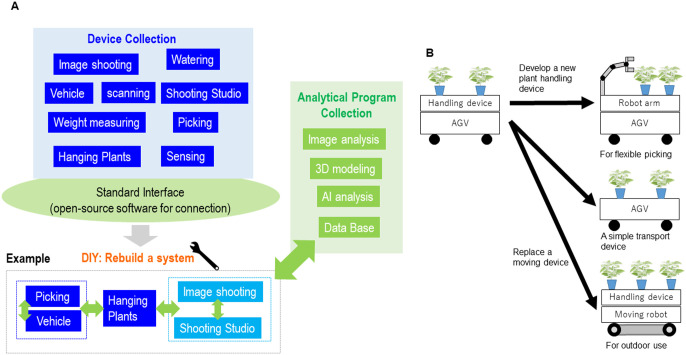
Technical elements required to realize DIY phenotyping.

We considered that the ideal DIY phenotyping system would 1) provide devices with unique functions that can be connected on a common platform, and 2) allow users to combine the devices like Lego blocks to rebuild the system according to their purposes (Fig. 6A). DIY phenotyping should not require users to have full knowledge of the devices or software programs. For DIY phenotyping to thrive, the prior existence of hardware and software that can be reconfigured by users without extensive knowledge of computer programming is essential. Another critical point for the advance of DIY phenotyping is that designed modules dissect functionality into multiple separate parts, to allow for “mix and match” development. In this study, we developed two devices independently. The running AGV device can be used for other purposes, for example, delivering sensors or cameras in front of the plants. It also can be used in agriculture, such as for transporting harvested plants from a cultivation area to a packing area. Likewise, the plant-handling device can be mounted to other types of mobile units for other situations (Fig. 6B). The value of such devices is increased by allowing for different combinations of devices.

The most effective approach to realize DIY phenotyping is to assemble commercial products and already available programs to build the system required by the experiment design envisioned by the users. In this system development, we used a commercial AGV kit for automatic driving, which enabled us to build a system without in-depth knowledge of AGV technology, simply by assembling the kit according to the manufacturer’s instructions. This meant that we were able to reduce the cost of building the mobile equipment. Use of commercial products as parts of the phenotyping system is also effective to keep robustness and high accuracy in DIY phenotyping. Commercial products have generally been sufficiently tested and improved to ensure that the product has a certain level of robustness and accuracy. On the other hand, fully self-designed and self-made products often do not have sufficient operational verification because this testing adds significant cost. In this study, we were able to guarantee the robustness and accuracy of the system for running by using the commercial AGV kit.

Of the two main tasks performed in plant phenotyping, conveyance, and measurement, the latter is easier to realize by DIY phenotyping. This is because many types of component products for measurement, such as sensors and microcomputer devices, are commercially available at reasonable cost, and information is far more abundant for programs that control those devices than for the systems needed for conveyance. DIY construction of automated devices for conveyance is more difficult because available commercial products are fewer and require higher levels of knowledge to employ. We developed our plant conveyance system with the aim of creating a module that users can choose as a transport device when assembling their own systems for DIY plant phenotyping. The AGV conveyance system we have developed will be disseminated through the Kazusa lab (https://www.kazusa.or.jp/kazusalab). In addition, people with more technical skills can build devices and programs with reference to the structures of devices and programs described in this manuscript to fit their own purposes. As with the AGV, several types of autonomous mobile robots, such as drones and carts, have been researched and developed and made available to users in recent years. It is expected that diverse types of autonomous mobile robots will contribute to further advances in DIY phenotyping.

### The key to DIY Plant phenotyping

**Fig. 7.**
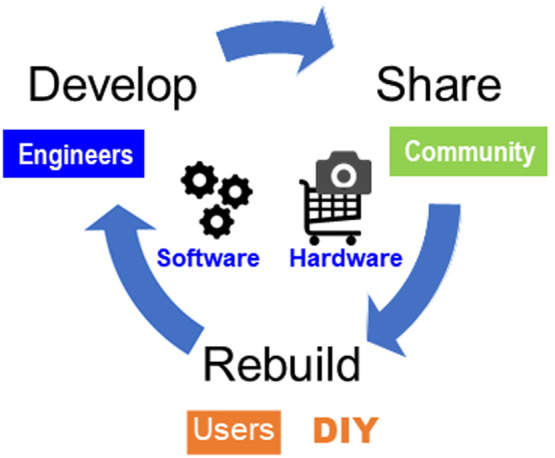
A sample action cycle for developing DIY phenotyping.

Providing devices and software to fit DIY phenotyping is important; however, it is not sufficient for popularizing it. For example, when a user intends to construct a certain system by DIY phenotyping, it is necessary to concretize the concept, create a system design, choose the optimal devices, and assemble them. These tasks are often difficult for a user without sufficient knowledge of plant phenotyping or for whom establishing the system would requires a great expenditure of effort and time. The key to DIY phenotyping is to share the information needed to build the system as well as making available the modules and components that comprise the system. This process can be described as a cycle of three actions: develop, share, and rebuild (Fig. 7). The cycle is also effective to increase the accuracy of modules developed by DIY phenotyping. Research communities and academic societies such as IPPN (International Plant Phenotyping Network, https://www.plant-phenotyping.org/) play important roles as venues to exchange information to support DIY phenotyping. In addition, optimization of technology is most efficiently achieved when engineers and users work together in the development process.

Open-source technology is another important concept in realizing DIY phenotyping. Open-source technology allows the sharing of hardware designs (CAD data, electronic circuit diagrams, etc.) and software programs, leading to the reuse of technologies developed by others. It also has the advantage of enhancing understanding of the structure inherent in the hardware or software. The idea of open-source technologies has become increasingly popular in software development, and several open-source software systems or platforms have been made public for image analysis (Hartmann *et al.* 2011, Knecht *et al.* 2016, Minervini *et al.* 2017, Slovak *et al.* 2014), for image acquisition hardware and analytical software (Czedik-Eysenberg *et al.* 2018), for deep learning (Ubbens and Stavness 2018) and for IoT sensor-based data management (Reynolds *et al.* 2019). Another concrete way to promote DIY phenotyping through open-source technology is to “farm out” the development of necessary components through collaborations on a specific project, as is being done by Kickstarter (https://www.kickstarter.com). The system developed in this study has not yet been developed for open-source publication, but we intend to revise our devices as open source in the near future.

### Conclusion

Many of the necessary elements for DIY phenotyping described in this manuscript have not yet been realized. However, in the development of technologies for new fields, it is important to propose an overall concept, and allow the elements to be filled in like pieces of a puzzle. The plant conveyance system described in this paper was developed as one of the pieces to fill in such an overall concept. We hope to advance discussions in the plant measurement community not only about the technology itself, but also about the philosophy behind its development.

## Author Contribution Statement

TT designed and fabricated the system and wrote the paper; KK, TH and HT performed the operational testing of the prototype and developed system; DI provided the basic idea of the system; SI managed the study and wrote the paper.

## Figures and Tables

**Table 1.** Advantages and disadvantages of transport systems in indoor plant phenotyping

	Belt conveyor	Mobile cart	Drone
Setting cost	High	Middle	Low
Maintenance cost	High	Low	Low
Ease of layout changes	Hard	Middle	Easy
Weight and number limit for items to be carried	Few	Middle	High
Robustness of transport	High	Middle	Low
References	Arend *et al.* 2016, Hairmansis *et al.* 2014, Klukas *et al.* 2014, Mazis *et al.* 2020, Pratap *et al.* 2015	Xiang *et al.* 2020, this study	Frangulea *et al.* 2021
